# Technical feasibility of constant-load and high-intensity interval training for cardiopulmonary conditioning using a re-engineered dynamic leg press

**DOI:** 10.1186/s42490-019-0025-9

**Published:** 2019-10-03

**Authors:** Farouk Chrif, Tobias Nef, Kenneth J Hunt

**Affiliations:** 1 Institute for Rehabilitation and Performance Technology, Division of Mechanical Engineering, Department of Engineering and Information Technology, Bern University of Applied Sciences, Burgdorf, CH-3400 Switzerland; 2 Gerontechnology and Rehabilitation Research Group, ARTORG Center for Biomedical Engineering Research, University of Bern, Bern, CH-3008 Switzerland

**Keywords:** Cardiorespiratory fitness, Resistance training, Exercise test, Exercise, High-intensity interval training, Heart rate, Anaerobic threshold, Feedback, Sensory

## Abstract

**Background:**

Leg-press devices are one of the most widely used training tools for musculoskeletal strengthening of the lower-limbs, and have demonstrated important cardiopulmonary benefits for healthy and patient populations. Further engineering development was done on a dynamic leg-press for work-rate estimation by integrating force and motion sensors, power calculation and a visual feedback system for volitional work-rate control. This study aimed to assess the feasibility of the enhanced dynamic leg press for cardiopulmonary exercise training in constant-load training and high-intensity interval training. Five healthy participants aged 31.0±3.9 years (mean ± standard deviation) performed two cardiopulmonary training sessions: constant-load training and high-intensity interval training. Participants carried out the training sessions at a work rate that corresponds to their first ventilatory threshold for constant-load training, and their second ventilatory threshold for high-intensity interval training.

**Results:**

All participants tolerated both training protocols, and could complete the training sessions with no complications. Substantial cardiopulmonary responses were observed. The difference between mean oxygen uptake and target oxygen uptake was 0.07±0.34 L/min (103 ±17%) during constant-load training, and 0.35±0.66 L/min (113 ±27%) during high-intensity interval training. The difference between mean heart rate and target heart rate was −7±19 bpm (94 ±15%) during constant-load training, and 4.2±16 bpm (103 ±12%) during high-intensity interval training.

**Conclusions:**

The enhanced dynamic leg press was found to be feasible for cardiopulmonary exercise training, and for exercise prescription for different training programmes based on the ventilatory thresholds.

## Background

It has been shown that exercise training has numerous health benefits both for healthy individuals and for patient populations, and physical inactivity can increases the risks of chronic diseases [1–3]. These health benefits are often associated with higher cardiorespiratory fitness levels (peak oxygen uptake $\dot {V}\mathrm {O}_{2\text {peak}}$, peak heart rate HR_peak_), which can be improved and increased by regular exercise training. A complete training programme should include aerobic exercise, muscular fitness, and flexibility exercise, and it is important to be well designed following exercise prescription guidelines (frequency, intensity, and duration) to meet an individual’s health and physical fitness goals. The recommended exercise prescription for most adults is an accumulation of at least 150 min.wk^−1^ at moderate intensity, or 75 min.wk^−1^ at vigorous intensity [4].

The most widely adopted exercise training techniques in clinical and sports environments are constant-load training (CLT) and high-intensity interval training (HIIT). A CLT programme consists of a continuous training phase at light (57-63% of HR_peak_, or 37-45% of $\dot {V}\mathrm {O}_{2\text {peak}}$) to moderate intensity (64-76% of HR_peak_, or 46-63% of $\dot {V}\mathrm {O}_{2\text {peak}}$) for at least 30 min [4]. CLT is often recommended for patient populations, because of high safety and tolerability [5]. On the other hand, HIIT can be defined as repeated short bouts of vigorous intensity training (77-95% of HR_peak_, or 64-90% of $\dot {V}\mathrm {O}_{2\text {peak}}$). The duration of the intervals can vary from 5 s to 8 min, depending on the nature of the exercise and the training device used [4, 6]. HIIT has been proven to produce improvements in physiological power for both healthy [7, 8] and patient [9, 10] populations, and it is often considered as a time-efficient training strategy, where some studies reported that programmes as short as two weeks’ duration could elicit substantial increases in $\dot {V}\mathrm {O}_{2\text {peak}}$ [8, 11].

The prescription of a correct exercise intensity is important and has to be tailored individually depending on the person’s capacity. It is either related to a percentage of peak capacity ($\dot {V}\mathrm {O}_{2\text {peak}}$, HR_peak_) or linked to the ventilatory thresholds (first ventilatory threshold VT1, and second ventilatory threshold VT2) [12]. The use of ventilatory thresholds for exercise prescription is recommended [13–15] because, unlike the maximal capacity percentage, ventilatory thresholds are independent of participants’ motivation [16] and the duration of the exercise testing protocol [17]. It was reported previously that a dynamic leg-press is feasible for incremental cardiopulmonary exercise testing, and the substantial cardiopulmonary responses allowed the identification of peak and sub-maximal parameters with a high rate of success [18].

Leg-press training devices allow the performance of resistive exercise, where eccentric and concentric work regimes are combined [18, 19]; this has been proven to provide important physiological benefits [20–22]. They are mainly used for musculoskeletal conditioning [23], and showed potential for neuromuscular rehabilitation applications [24]. A previous study evaluated changes in $\dot {V}\mathrm {O}_{2\text {peak}}$ and found that a leg press exercise training programme elicits larger increases in $\dot {V}\mathrm {O}_{2\text {peak}}$ when tested on the leg press than when tested on a cycle or a treadmill [25]. Another study reported that leg press exercise elicits cardiovascular benefits when used as resistance training in cardiac patients [26].

To facilitate and to promote active participation during training sessions, further engineering development was necessary on a dynamic leg-press for work-rate estimation by integrating force sensors behind the footplates, motion sensors in the rotation axis of the pedals, power calculation and a visual feedback system. These engineering modifications facilitated accurate work rate calculation, and allowed participants to see their exercise work rate and adjust their volitional effort to meet the target work rate. This approach was previously shown to be feasible for incremental cardiopulmonary exercise testing in healthy participants [18].

The aim of this study was to assess the feasibility of the re-engineered dynamic leg press for cardiopulmonary exercise training (CLT and HIIT programmes) in healthy males. Feasibility assessment considered: (i) technical implementation, (ii) acceptability and (iii) responsiveness.

## Results

Individual outcome data from the 5 participants in the case series are presented in Table 1 and illustrated in Figs. 3, 4, 5, 6, and 7. The data show that in all cases highly accurate work rate control was achieved for all CLT and HIIT target profiles (Figure parts (g) and (h)).

**Table 1:** Cardiopulmonary outcomes

Participant	1	2	3	4	5	mean ± SD
CLT						
$\dot {V}\mathrm {O}_{2}$	1.54	1.18	1.32	1.36	2.88	1.66 ±0.70
% of $\dot {V}\mathrm {O}_{2\text {peak}}$	52	42	40	39	68	48 ±12
HR	114	121	137	91	156	124 ±24
% of HR_peak_	76	63	72	56	83	70 ±11
HIIT						
$\dot {V}\mathrm {O}_{2}$	3.67	2.51	2.62	1.93	4.21	2.99 ±0.93
% of $\dot {V}\mathrm {O}_{2\text {peak}}$	124	90	79	55	100	90 ±26
HR	164	173	177	118	177	162 ±25
% of HR_peak_	109	90	92	72	94	91 ±13

For the CLT, $\dot {V}\mathrm {O}_{2}$ and HR are the average oxygen uptake and heart rate obtained during the active phases

For the HIIT, $\dot {V}\mathrm {O}_{2}$ and HR are the average of the five peak values obtained from each interval

**Table 2:** Participants’ characteristics and target data

Participant		1		2		3		4		5		mean ± SD
Characteristics												
Age range (yr)		30-35		30-35		25-30		25-30		30-35		31.0 ±3.9
Body mass (kg)		86		70		81		80		73		78.0 ±6.4
Height (m)		1.88		1.72		1.81		1.83		1.82		1.81 ±0.06
BMI (kg/ *m*^2^)		24.3		23.7		24.7		23.9		22.0		23.7 ±1.0
Incremental test outcomes												
oxygen uptake (L/min)												
$\dot {V}\mathrm {O}_{2\text {peak}}$		2.95		2.79		3.32		3.49		4.22		3.34 ±0.56
$\dot {V}\mathrm {O}_{2\text {VT}1}$		1.57		1.05		1.51		1.56		2.26		1.59 ±0.43
$\dot {V}\mathrm {O}_{2\text {VT}2}$		2.35		2.32		2.52		2.38		3.64		2.64 ±0.56
heart rate (bpm)												
HR_peak_		150		192		193		163		188		177 ±20
HR_VT1_		115		138		144		122		135		131 ±12
HR_VT2_		136		176		172		133		171		158 ±21

During the CLT, the overall difference, averaged across all participants, between mean steady-state oxygen uptake $\dot {V}\mathrm {O}_{2}$ and $\dot {V}\mathrm {O}_{2\text {VT}1}$ was $\Delta \dot {V}\mathrm {O}_{2\text {VT}1}=0.07 \pm 0.34$ L/min (103 ±17%, relative to target value) (mean ± SD). The overall difference between mean steady-state heart rate HR and HR_VT1_ was *Δ*HR_VT1_=−7±19 bpm (94 ±15%).

During the HIIT, the overall difference between the mean of the highest oxygen uptake values during each high-intensity phase $\dot {V}\mathrm {O}_{2}$ and $\dot {V}\mathrm {O}_{2\text {VT}2}$ was $\Delta \dot {V}\mathrm {O}_{2\text {VT}1}=0.35 \pm 0.66$ L/min (113 ±27%). The overall difference between the mean of the highest heart rate values HR and HR_VT2_ was *Δ*HR_VT2_=4±16 bpm (103 ±12%).

The DLP is deemed to be feasible for performing cardiopulmonary exercise training, since the formal feasibility assessment criteria were evaluated as follows:

(i) Implementation: The augmentation of the DLP with work rate estimation and visual feedback enabled the successful implementation of the training programmes (viz. CLT and HIIT).
(ii) Acceptability: All participants tolerated both training protocols, and could complete the training sessions with no complications.
(iii) Responsiveness: Substantial cardiopulmonary responses were observed for both CLT and HIIT: for CLT, the mean steady-state oxygen uptake $\dot {V}\mathrm {O}_{2}$ was 48% of $\dot {V}\mathrm {O}_{2\text {peak}}$ and mean steady-state heart rate HR was 70% of HR_peak_; for HIIT, the mean of the highest oxygen uptake values was 90% of $\dot {V}\mathrm {O}_{2\text {peak}}$ and the mean of the highest heart rate values was 91% of HR_peak_.

## Discussion

The aim of this study was to assess the feasibility of a dynamic leg press for cardiopulmonary exercise training (CLT and HIIT programmes) in healthy males. Feasibility assessment considered: (i) technical implementation, (ii) acceptability and (iii) responsiveness.

The DLP is deemed feasible for performing cardiopulmonary exercise training. Regarding the implementation, the augmentation of the DLP with work rate estimation and visual feedback enabled the implementation of the exercise training programmes (viz. CLT and HIIT). In term of acceptability and responsiveness, all participants could understand the task and adapt their volitional leg effort to keep to the target work rate. All participants could complete the training sessions with no complications, and substantial cardiopulmonary responses were observed for both CLT and HIIT.

Four participants performed the training sessions for both CLT and HIIT based on their target $\dot {V}\mathrm {O}_{2\text {VT}1}$ and $\dot {V}\mathrm {O}_{2\text {VT}2}$ obtained from the previous incremental tests [18]. One participant (participant 1) could not complete the training sessions based on the target outcomes from the incremental test [18]. It was suspected that these outcomes were overestimated, therefore this participant had to re-perform the incremental test where the outcomes were found to be lower than those obtained from the first incremental test.

For two participants (participants 1 and 5), the values obtained were higher than the recommended range of HIIT (vigorous intensity), and corresponded to “near maximal to maximal” intensity (Table 1). As for the CLT, only participant 5 had outcomes higher than the recommended “light to moderate intensity” (Table 1).

Considering $\dot {V}\mathrm {O}_{2}$ in the HIIT protocol, it was observed that participants 1 and 5 performed at intensities which correspond to 124% and 100% of $\dot {V}\mathrm {O}_{2\text {peak}}$ respectively, while, surprisingly, participant 4 performed at only 55% of his $\dot {V}\mathrm {O}_{2\text {peak}}$, which is described as “moderate intensity”. The high $\dot {V}\mathrm {O}_{2}$ obtained from participants 1 and 5 may be due to the $\dot {V}\mathrm {O}_{2\text {peak}}$ obtained from the incremental exercise test being due to muscle fatigue after ∼10 min of exercise [18] and not aerobic exhaustion. During the HIIT bouts, the participants tried to reach the target intensity as quickly as possible and kept exercising for a relatively short duration (2 min) at their VT2 which is near to maximal capacity [12], thus high $\dot {V}\mathrm {O}_{2}$ values were elicited. On the other hand, participant 4, who had low cardiovascular outcomes during CLT and HIIT, seemed to be unfamiliar with this kind of exercise (resistive leg exercise). In general, the fact that the participant is not familiar with the type of exercise impairs the result during the exercise test, and the calculated work load is often too low [27].

The test-retest reliability and repeatability of incremental testing using the DLP has not been evaluated, and the predicted peak work rate during the incremental test was adapted from a method developed for a cycle ergometer [28]. As a complement to the present assessment of the DLP for exercise training, future work should include the evaluation of test-retest reliability of cardiopulmonary exercise testing using the DLP, the establishment of a method to predict peak work rate during incremental tests, and the investigation of physiological adaptations to long term training programmes for healthy and patient populations.

## Conclusions

The dynamic leg press was found to be feasible for cardiopulmonary exercise training, and for exercise prescription for different training programmes (viz. CLT and HIIT) based on the ventilatory thresholds.

## Methods

### Research participants and study design

This feasibility study was reviewed and approved by the Ethics Review Board of the Canton of Bern in Switzerland (Kantonale Ethikkommission Bern, KEK. Ref. No. 2016-01502). Written informed consent was obtained from all participants.

Five healthy males (age 31.0 ±3.9), all non-smokers with no known cardiovascular, pulmonary or musculoskeletal problems participated (Table 2). The five participants had been recruited among participants in a previous study, where they performed incremental cardiopulmonary exercise tests on the same training device [18]. The two studies were separated by 7 months. Peak cardiopulmonary outcomes ($\dot {V}\mathrm {O}_{2\text {peak}}$ and HR_peak_), and sub-maximal cardiopulmonary thresholds (first and second ventilatory thresholds $\dot {V}\mathrm {O}_{2\text {VT}1}$, $\dot {V}\mathrm {O}_{2\text {VT}2}$, and heart rates at the first and second ventilatory thresholds HR_VT1_, HR_VT2_) obtained from the previous study were used for the individual training prescription and analysis (Table 2).

Each participant carried out 2 formal training sessions: (i) a constant-load test (CLT), where participants trained for 30 min at their personal *P*_VT1_ (work rate that corresponds to $\dot {V}\mathrm {O}_{2\text {VT}1}$ obtained from the incremental test); (ii) a high-intensity interval training (HIIT) session, where participants performed 5 high intensity intervals at their personal *P*_VT2_ (work rate that corresponds to $\dot {V}\mathrm {O}_{2\text {VT}2}$ obtained from the incremental test). Training sessions were separated by at least 48 h. Participants were required to avoid strenuous activity within the 24 h prior to each formal test session, to refrain from caffeine for 12 h before, and not to consume a large meal within 3 h prior to testing.

**Fig. 1:**
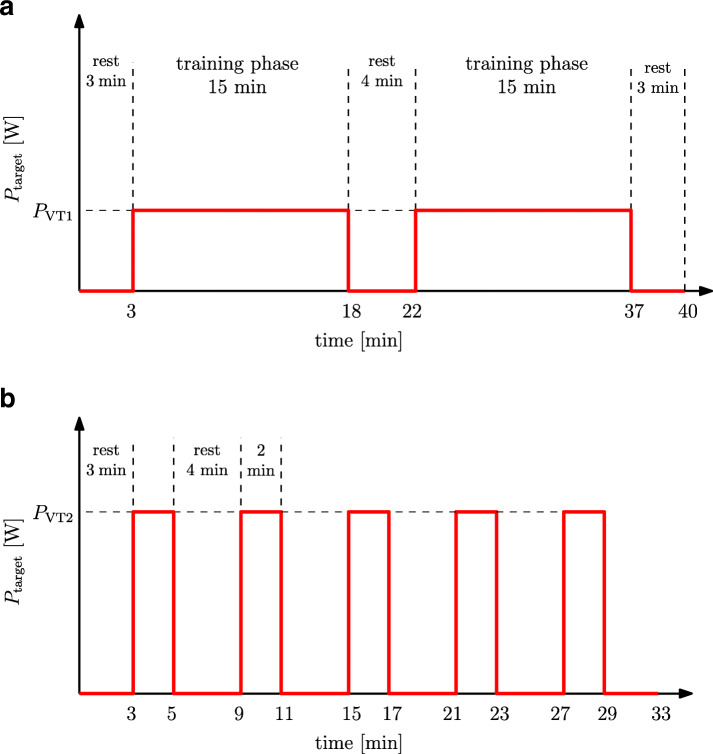
Exercise testing protocols. *P*_target_: target work rate. **a** CLT protocol; **b** HIIT protocol

The CLT protocol (Fig. 1a) consisted of 5 stages: a 3-min recorded rest; 2 training phases of 15 min each separated by a 4-min rest period; and a 3-min recorded rest. During the training phases participants were instructed to follow a predefined target work rate *P*_VT1_ which corresponds to their individual $\dot {V}\mathrm {O}_{2\text {VT}1}$.

The HIIT protocol (Fig. 1b) started with a 3-min recorded rest, then 5 training intervals of 2 min at a work rate *P*_VT2_ corresponding to the individual $\dot {V}\mathrm {O}_{2\text {VT}2}$, interspersed by 4-min rest periods. The HIIT session was ended by a 3-min recorded rest. For both CLT and HIIT, participants performed 2 min of unrecorded warm-up at low intensity prior to the training session.

### Equipment

**Fig. 2:**
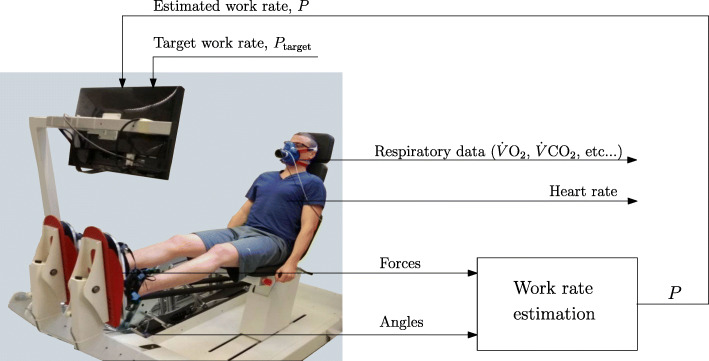
Dynamic leg press augmented with work rate estimation and visual feedback. (adapted from [18])

A commercial dynamic leg press exercise device (Allegro, Dynamic Devices AG, Switzerland) was used in this study. The dynamic leg press is pneumatically-actuated and was augmented with force sensors in the foot plates, angle sensors at the rotation axis of the pedal, a work rate estimation algorithm, and a visual feedback (Fig. 2).

Respiratory variables and heart rate were monitored and recorded using a breath-by-breath cardiopulmonary monitoring system (Metamax 3B, Cortex Biophysik GmbH, Germany). Pressure, volume and gas concentration were calibrated prior to each test according the manufacturer’s instructions: pressure was calibrated using a certified atmospheric pressure device; volume using a 3 L syringe; and gas concentrations were calibrated using ambient air and a precision gas mixture (15% O_2_, 5% CO_2_). Heart rate was recorded using a chest belt (T34, Polar Electro Oy, Finland). Analysis of the cardiopulmonary data was done using the proprietary software associated with the breath-by-breath system (Metasoft, version 3.9.9 SR5).

**Fig. 3:**
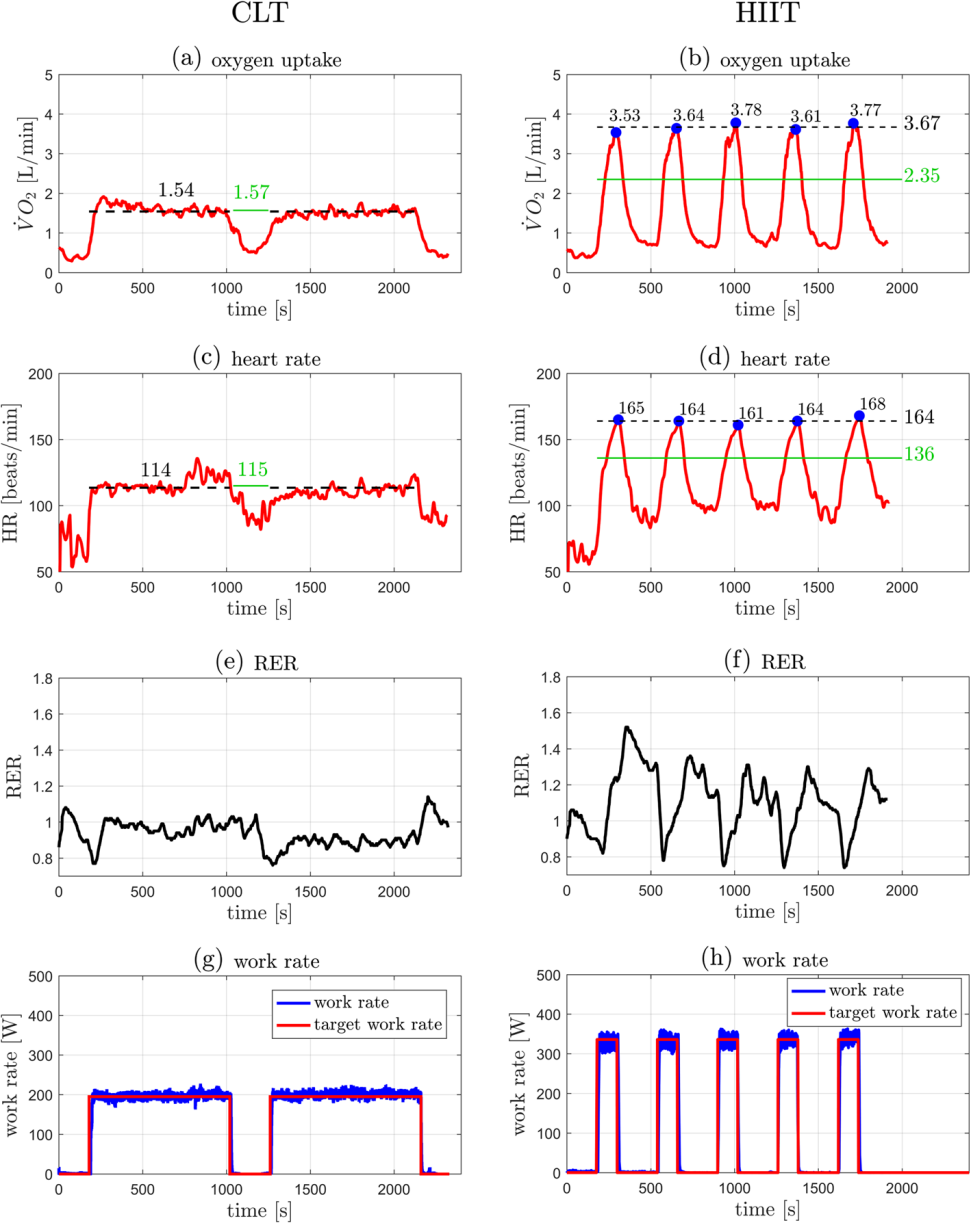
Original data records from participant 1. Left: CLT, Right: HIIT. (**a**,**b**) Oxygen uptake $\dot {V}\mathrm {O}_{2}$. (**c**,**d**) Heart rate HR. (**e**,**f**) Respiratory exchange ratio (RER). (**g**,**h**) Target and measured work rates (*P*_target_, *P*). Parts (**a**)–(**d**): the green line indicates the target values, $\dot {V}\mathrm {O}_{2\text {VT}1}$ for the CLT and $\dot {V}\mathrm {O}_{2\text {VT}2}$ for the HIIT (Table 2); the dashed black line indicates the measured mean value, the steady state mean value for CLT, and the average of the five peak values for HIIT (Table 1). Parts (**b**),(**d**): the blue dots indicate the peak values of each interval during HIIT

### Work rate estimation and targets

During the training sessions, the participant’s feet were fixed on footplates, and his work rate was estimated using force and velocity data (Fig. 2). The work rate estimation algorithm was implemented in Matlab/Simulink (The MathWorks, Inc., USA). The subject’s work rate (*P*) was estimated using force and velocity data provided by the force and angel sensors, and using the following equation:

1 $$ \begin{aligned} P &= P_{\mathrm{l}} + P_{\mathrm{r}} \\ &= F_{\mathrm{l}} R \dot{\theta}_{\mathrm{l}} + F_{\mathrm{r}} R \dot{\theta}_{\mathrm{r}} \end{aligned}  $$

**Fig. 4:**
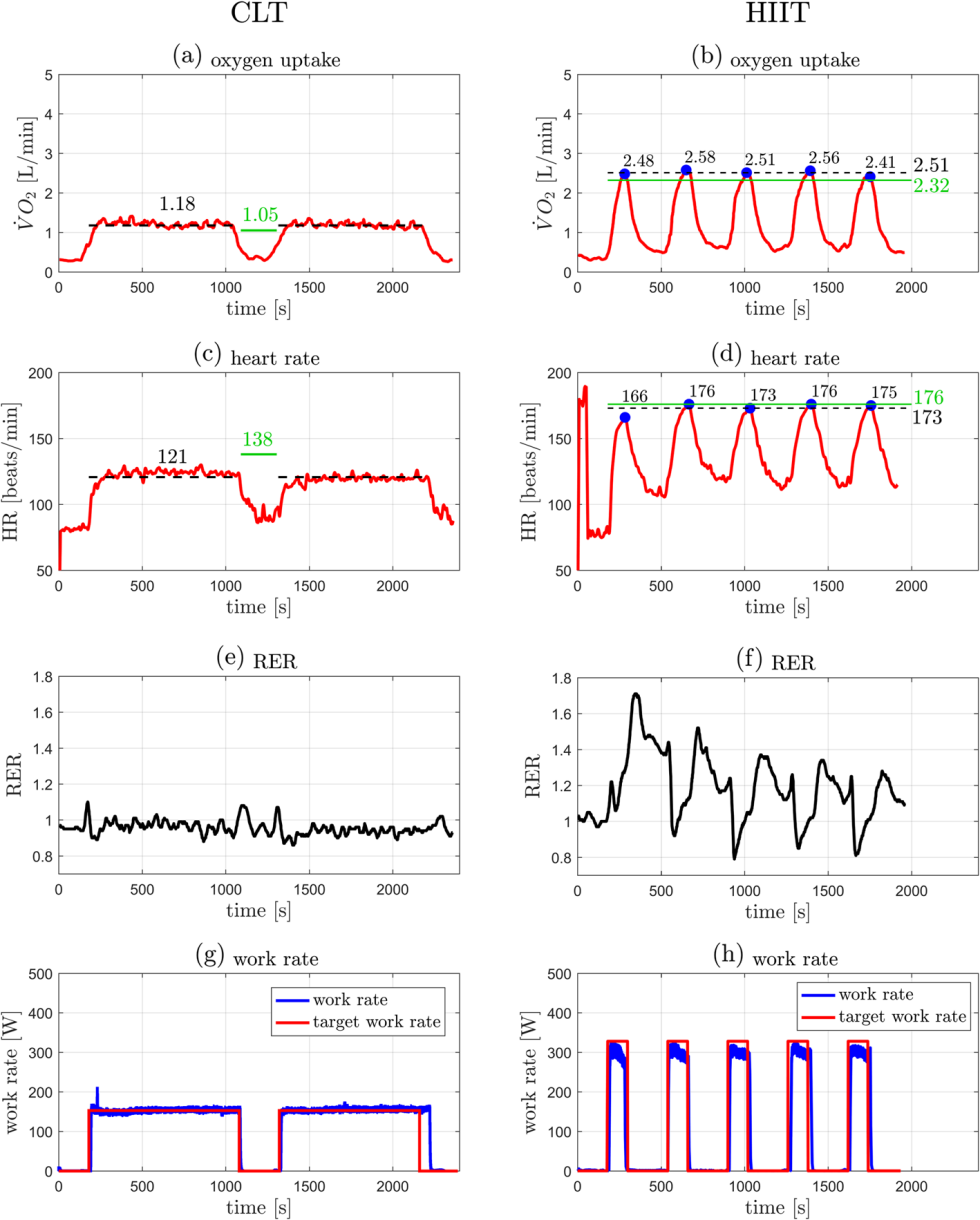
Original data records from participant 2. Left: CLT, Right: HIIT. (**a**,**b**) Oxygen uptake $\dot {V}\mathrm {O}_{2}$. (**c**,**d**) Heart rate HR. (**e**,**f**) Respiratory exchange ratio (RER). (**g**,**h**) Target and measured work rates (*P*_target_, *P*). Parts (**a**)–(**d**): the green line indicates the target values, $\dot {V}\mathrm {O}_{2\text {VT}1}$ for the CLT and $\dot {V}\mathrm {O}_{2\text {VT}2}$ for the HIIT (Table 2); the dashed black line indicates the measured mean value, the steady state mean value for CLT, and the average of the five peak values for HIIT (Table 1). Parts (**b**),(**d**): the blue dots indicate the peak values of each interval during HIIT

where *P*_l_ and *P*_r_ are the work rates of the left and right legs, *F*_l_ and *F*_r_ are the forces applied by the left and right legs, *R* the radius of the pedal, and $\dot {\theta }_{\mathrm {l}}$ and $\dot {\theta }_{\mathrm {r}}$ are the left and right pedal’s angular velocity [18]. Subjects were asked to adjust their volitional effort to maintain the target work rate.

**Fig. 5:**
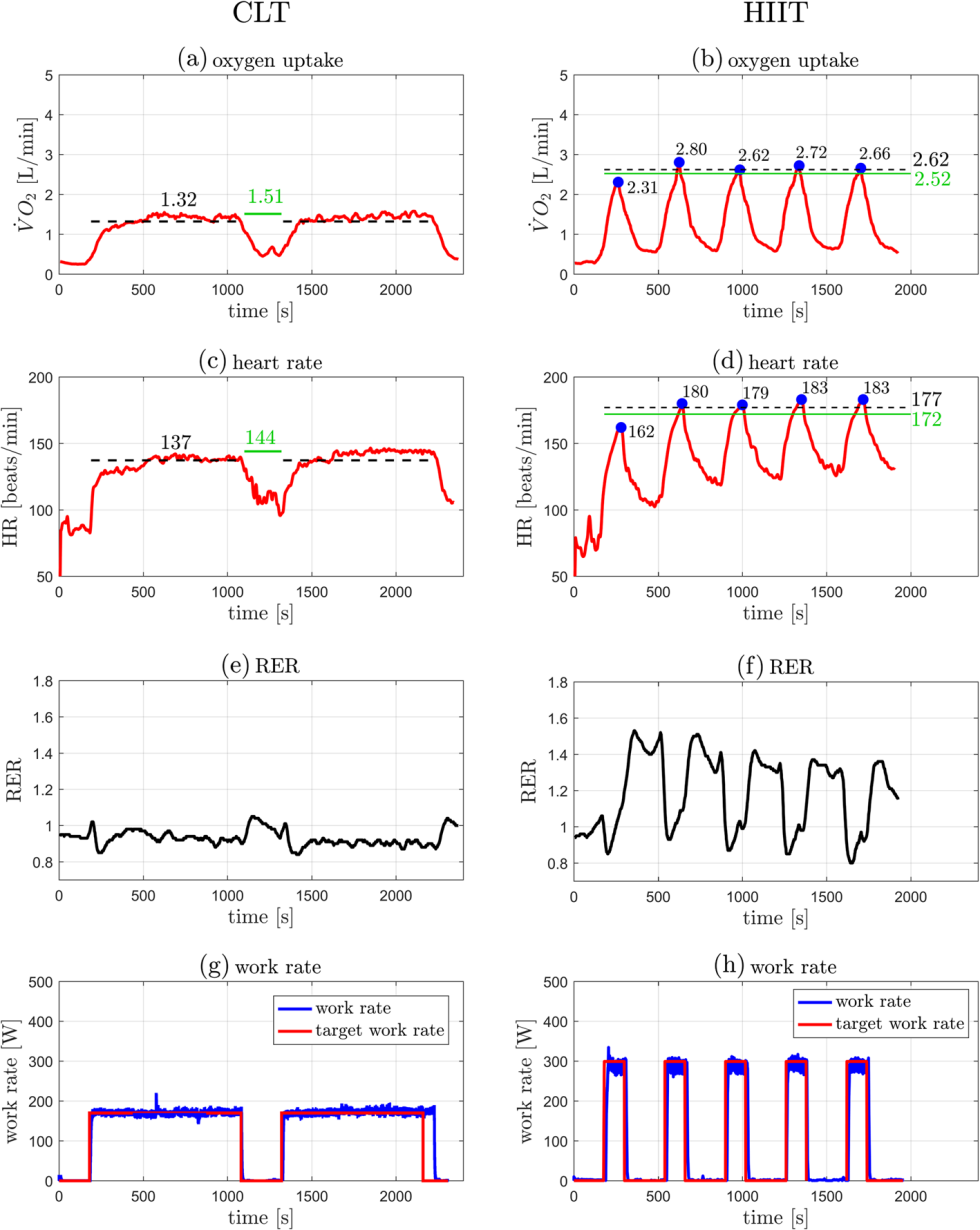
Original data records from participant 3. Left: CLT, Right: HIIT. (**a**,**b**) Oxygen uptake $\dot {V}\mathrm {O}_{2}$. (**c**,**d**) Heart rate HR. (**e**,**f**) Respiratory exchange ratio (RER). (**g**,**h**) Target and measured work rates (*P*_target_, *P*). Parts (**a**)–(**d**): the green line indicates the target values, $\dot {V}\mathrm {O}_{2\text {VT}1}$ for the CLT and $\dot {V}\mathrm {O}_{2\text {VT}2}$ for the HIIT (Table 2); the dashed black line indicates the measured mean value, the steady state mean value for CLT, and the average of the five peak values for HIIT (Table 1). Parts (**b**),(**d**): the blue dots indicate the peak values of each interval during HIIT

Based on the personal sub-maximal outcomes obtained from the incremental tests performed in the previous study [18], CLT and HIIT protocols were set individually for each participant: for the CLT, the target work rate *P*_VT1_ is the work rate during the ramp phase of the incremental test at the time the first ventilatory threshold occurred. For the HIIT, the target work rate *P*_VT2_ is the work rate at the time of the second ventilatory threshold. Participants were required to adapt their volitional leg effort to keep the estimated work rate as close as possible to the target work rate, and to maintain a constant stepping cadence of 60 steps/min following the tones of a metronome.

**Fig. 6:**
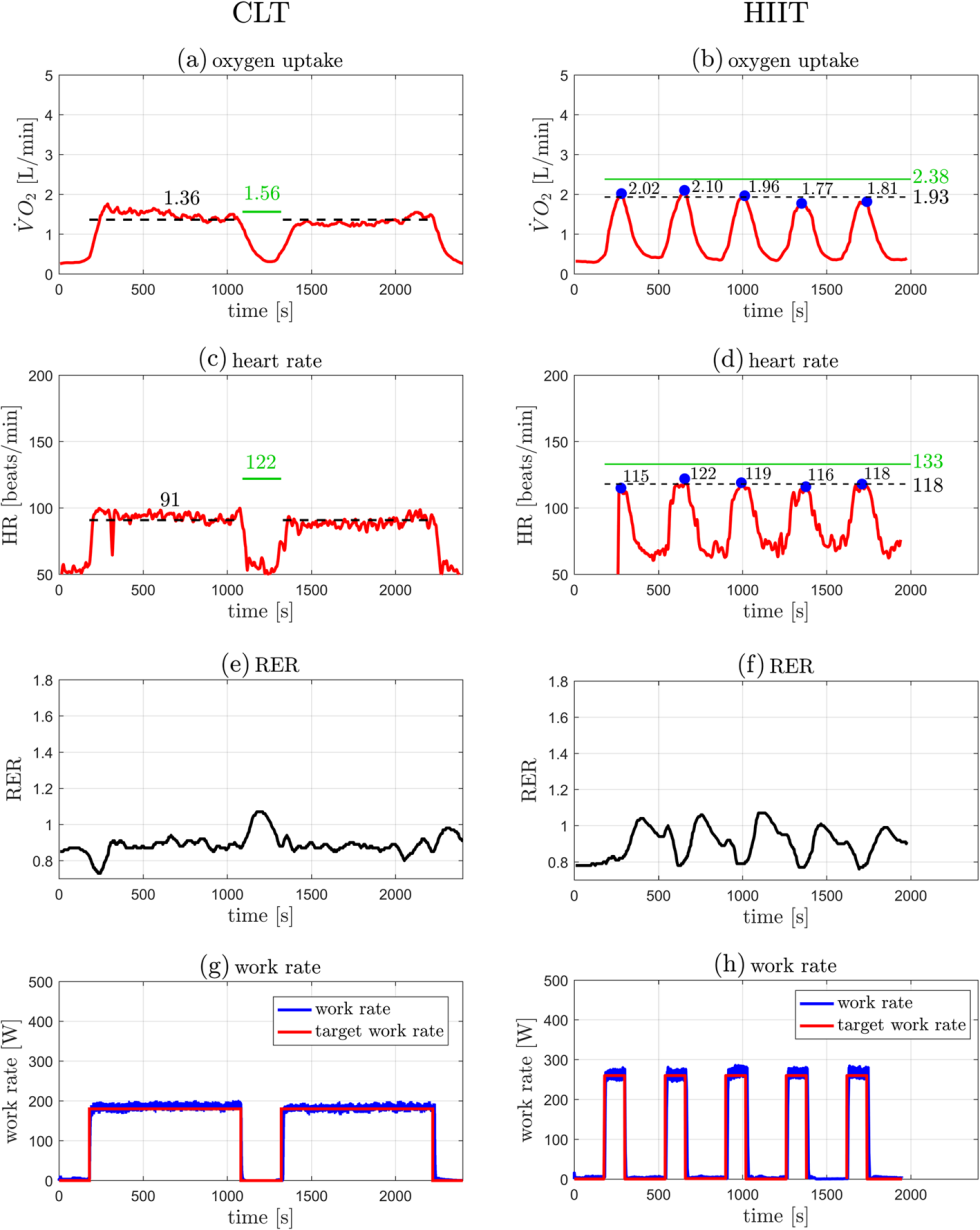
Original data records from participant 4. Left: CLT, Right: HIIT. (**a**,**b**) Oxygen uptake $\dot {V}\mathrm {O}_{2}$. (**c**,**d**) Heart rate HR. (**e**,**f**) Respiratory exchange ratio (RER). (**g**,**h**) Target and measured work rates (*P*_target_, *P*). Parts (**a**)–(**d**): the green line indicates the target values, $\dot {V}\mathrm {O}_{2\text {VT}1}$ for the CLT and $\dot {V}\mathrm {O}_{2\text {VT}2}$ for the HIIT (Table 2); the dashed black line indicates the measured mean value, the steady state mean value for CLT, and the average of the five peak values for HIIT (Table 1). Parts (**b**),(**d**): the blue dots indicate the peak values of each interval during HIIT

### Outcome measures and data analysis

For the CLT, outcome measures were steady-state levels of $\dot {V}\mathrm {O}_{2}$ and HR: $\dot {V}\mathrm {O}_{2}$ is the mean oxygen uptake obtained during the active phases with a 15-breath moving average. HR is the mean heart rate obtained during the active phases.

**Fig. 7:**
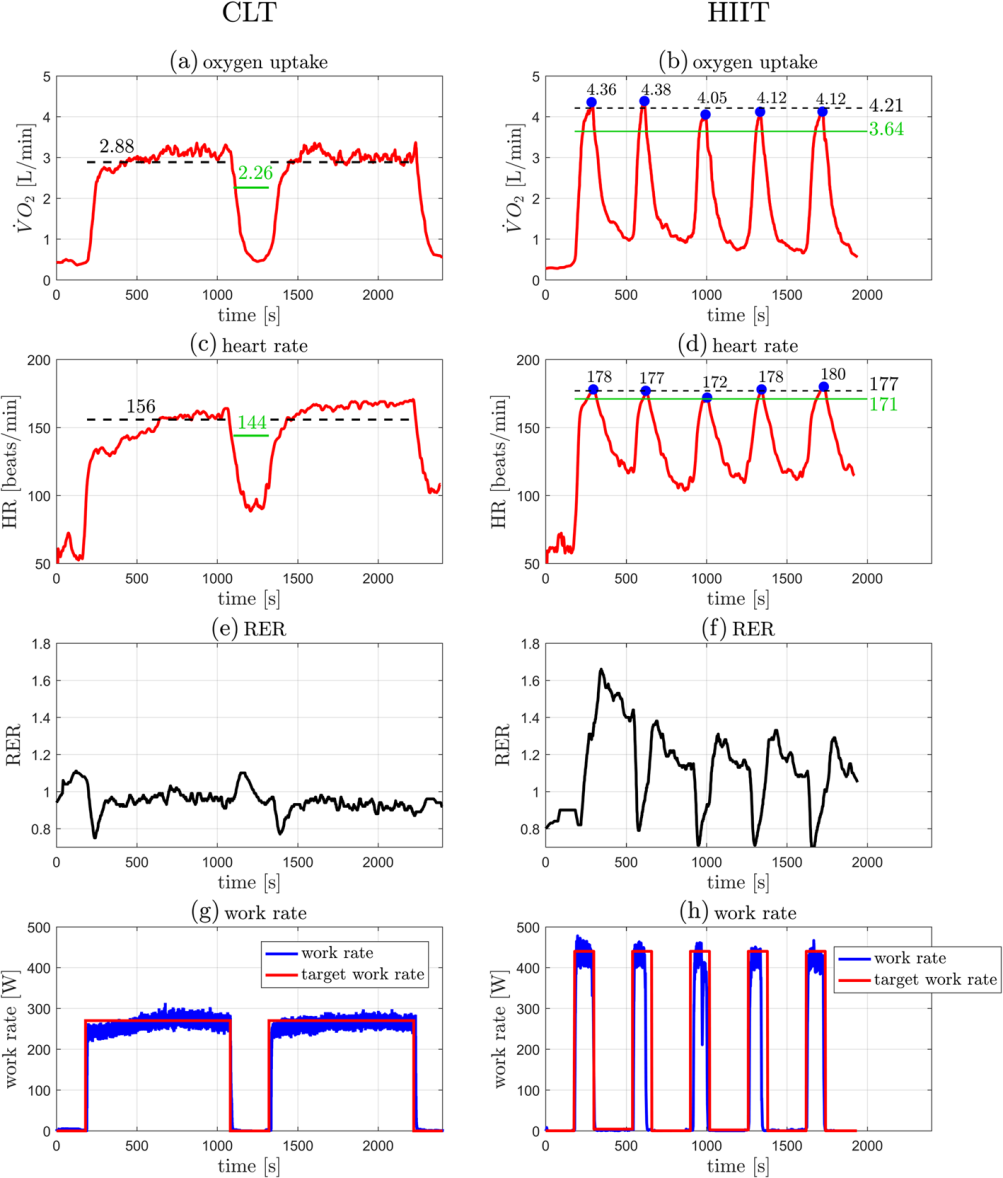
Original data records from participant 5. Left: CLT, Right: HIIT. (**a**,**b**) Oxygen uptake $\dot {V}\mathrm {O}_{2}$. (**c**,**d**) Heart rate HR. (**e**,**f**) Respiratory exchange ratio (RER). (**g**,**h**) Target and measured work rates (*P*_target_, *P*). Parts (**a**)–(**d**): the green line indicates the target values, $\dot {V}\mathrm {O}_{2\text {VT}1}$ for the CLT and $\dot {V}\mathrm {O}_{2\text {VT}2}$ for the HIIT (Table 2); the dashed black line indicates the measured mean value, the steady state mean value for CLT, and the average of the five peak values for HIIT (Table 1). Parts (**b**),(**d**): the blue dots indicate the peak values of each interval during HIIT

For the HIIT, outcome measures were the peak values obtained during the active intervals: $\dot {V}\mathrm {O}_{2}$ is the average of the five peak oxygen uptake values obtained from each interval, using a 15-breath moving average. HR is the average of the five peak heart rate values obtained from each interval. For both protocols, the respiratory exchange ratio $\text {RER} = \dot {V}\text {CO}_{2}/\dot {V}\mathrm {O}_{2}$ was recorded with a 15-breath moving average.

Descriptive analyses were performed on all variables. To assess the results obtained from the training sessions of each participant, comparison of training results and incremental test results were performed: for CLT, the mean values of steady-state levels were compared to the corresponding values of $\dot {V}\text {O}_{2\text {VT}1}$ obtained from the incremental test; for HIIT, means of the 5 highest values were compared to the corresponding values of $\dot {V}\mathrm {O}_{2\text {VT}2}$ obtained from the incremental test. All analyses and displays were performed using MetaSoft and Matlab.

Individual outcome measures were recorded for each participant and are presented in “Results” section (Figs. 3, 4, 5, 6, and 7). For the CLT protocol, the target values ($\dot {V}\mathrm {O}_{2\text {VT}1}$ and HR_VT1_) are presented with a green line, and the mean steady state value with a dashed black line; for the HIIT protocol, the green line represents the target values ($\dot {V}\mathrm {O}_{2\text {VT}2}$ and HR_VT2_) and the dashed black line represents the average of the 5 peak values obtained from the intervals (the blue dots).

### Feasibility assessment

The criteria employed for feasibility assessment of cardiopulmonary training using a dynamic leg-press were as follows [29]: (i) implementation - was the approach technically feasible for cardiopulmonary training?; (ii) acceptability - were the training protocols tolerable?; and (iii) responsiveness - was the approach able to provoke substantial cardiopulmonary responses in relation to individual peak values?

## Data Availability

The datasets used and/or analysed during the current study are available from the corresponding author on reasonable request.

## Abbreviations

bpm: beats per minute
CE: cycle ergometer
CI: confidence interval
DLP: dynamic leg press
HR: heart rate
HR_peak_: peak heart rate
MD: mean difference
*P*: work rate (synonymous with “
power”); P _ET_O_2_: partial pressure of end-tidal oxygen tension
P _ET_CO_2_: partial pressure of end-tidal carbon dioxide tension
*P*_target_: target work rate
RER: respiratory exchange ratio,$ ext {RER} riangleq \dot {V} ext {CO}_{2}/\dot {V}\mathrm {O}_{2}$
RER_peak_: peak respiratory exchange ratio
SD: standard deviation
$\dot {V}\mathrm {E}$: minute ventilation
$\dot {V}\mathrm {O}_{2 ext {VT}1}$: oxygen uptake at the first ventilatory threshold
$\dot {V}\mathrm {O}_{2 ext {VT}2}$: oxygen uptake at the second ventilatory threshold
$\dot {V}\mathrm {O}_{2 ext {peak}}$: peak oxygen uptake
$\dot {V}\mathrm {O}_{2}$: rate of oxygen uptake (referred to simply as “
oxygen uptake”); $\dot {V} ext {CO}_{2}$: rate of carbon dioxide output
*t*_ramp_: ramp duration
$\dot {V}\mathrm {E}/\dot {V}\mathrm {O}_{2}$: ventilatory equivalent of oxygen
$\dot {V}\mathrm {E}/\dot {V} ext {CO}_{2}$: ventilatory equivalent of carbon dioxide
VT1: first ventilatory threshold
VT2: second ventilatory threshold
